# MiR-29a Reduces TIMP-1 Production by Dermal Fibroblasts via Targeting TGF-β Activated Kinase 1 Binding Protein 1, Implications for Systemic Sclerosis

**DOI:** 10.1371/journal.pone.0115596

**Published:** 2014-12-30

**Authors:** Marzena Ciechomska, Steven O’Reilly, Monika Suwara, Katarzyna Bogunia-Kubik, Jacob M. van Laar

**Affiliations:** 1 Newcastle University, Musculoskeletal Research Group, Institute of Cellular Medicine, Newcastle upon Tyne, United Kingdom; 2 Newcastle University, Fibrosis Research Group, Institute of Cellular Medicine, Newcastle upon Tyne, United Kingdom; 3 L. Hirszferd Institute of Immunology and Experimental Therapy, Polish Academy of Science, Wroclaw, Poland; 4 University Medical Center Utrecht, Department of Rheumatology & Clinical Immunology, Utrecht, the Netherlands; Medical University of South Carolina, United States of America

## Abstract

**Background:**

Systemic sclerosis (SSc) is an autoimmune connective tissue disease characterised by skin and internal organs fibrosis due to accumulation of extra cellular matrix (ECM) proteins. Tissue inhibitor of metalloproteinases 1 (TIMP-1) plays a key role in ECM deposition.

**Aim:**

To investigate the role of miR-29a in regulation of TAB1-mediated TIMP-1 production in dermal fibroblasts in systemic sclerosis.

**Methods:**

Healthy control (HC) and SSc fibroblasts were cultured from skin biopsies. The expression of TIMP-1, MMP-1 and TGF-β activated kinase 1 binding protein 1 (TAB1) was measured following miR-29a transfection using ELISA, qRT-PCR, and Western Blotting. The functional effect of miR-29a on dermal fibroblasts was assessed in collagen gel assay. In addition, HeLa cells were transfected with 3′UTR of TAB1 plasmid cloned downstream of firefly luciferase gene to assess TAB1 activity. HC fibroblasts and HeLa cells were also transfected with Target protectors in order to block the endogenous miR-29a activity.

**Results:**

We found that TAB1 is a novel target gene of miR-29a, also regulating downstream TIMP-1 production. TAB1 is involved in TGF-β signal transduction, a key cytokine triggering TIMP-1 production. To confirm that TAB1 is a *bona fide* target gene of miR-29a, we used a TAB1 3′UTR luciferase assay and Target protector system. We showed that miR-29a not only reduced TIMP-1 secretion via TAB1 repression, but also increased functional MMP-1 production resulting in collagen degradation. Blocking TAB1 activity by pharmacological inhibition or TAB1 knockdown resulted in TIMP-1 reduction, confirming TAB1-dependent TIMP-1 regulation. Enhanced expression of miR-29a was able to reverse the profibrotic phenotype of SSc fibroblasts via downregulation of collagen and TIMP-1.

**Conclusions:**

miR-29a repressed TAB1-mediated TIMP-1 production in dermal fibroblasts, demonstrating that miR-29a may be a therapeutic target in SSc.

## Introduction

Systemic sclerosis (SSc) is an autoimmune disease characterized by increased production of collagen and other profibrotic factors, including tissue inhibitors of metalloproteinases (TIMPs).

TIMPs and matrix metalloproteinases (MMPs) are responsible for maintaining the balance between production and degradation of extracellular matrix proteins (ECM). Fibroblasts play an essential role during pathological remodelling of ECM by excessive deposition of collagen and TIMP-1 [1]. A previous study showed that TIMP-1 is increased in SSc sera and is excessively produced by fibroblasts isolated from the involved skin lesions of SSc patients [2], [3]. We have also demonstrated that monocytes from SSc patients produce more TIMP-1 following TLRs stimulation, thereby contributing to fibrosis development [4], [5]. In animal models, the serum level of TIMP-1 correlates with dexamethasone-induced liver fibrosis in rats. Likewise, TIMP-1 strongly promotes liver fibrosis development in CCL4-treated TIMP-1 transgenic mice [6], [7]. Furthermore, adenoviral overexpression of human recombinant TIMP-1 in murine fibroblasts increased their proliferation and differentiation toward pathogenic myofibroblast phenotype, which demonstrates an additional role of TIMP-1 beyond the inhibition of MMPs activity [8].

Recently, it has been shown that the fibrotic remodelling process is associated with an altered pattern of miRNAs expression. Micro RNAs (miRs) are small, noncoding (consisting of 19 to 22 nucleotides) RNAs that mostly bind to 3′UTRs of target mRNAs leading to gene silencing. Thus, miRs act as a fine-tuner of gene expression that negatively regulate between 25 and 60 percent of human protein-coding genes [9]. Furthermore, expression of miRs can be altered under conditions of pathophysiological stress or disease, allowing miRs to be important biomarkers and attractive candidates for therapeutic manipulation [10], [11]. Recent studies have shown that miRs are involved in pathological fibrosis in many organs including lung, heart, kidney and skin [12]–[14]. In particular, it has been demonstrated that the family of miR-29 is a key regulator of skin fibrosis [15], [16]. The miR-29 family consists of three members (miR29-a, -b, -c) which bind to an identical ‘seed’ sequence of transcript, therefore allowing the targeting of the same genes [12]. Various computational algorithms predicted that miR-29 can target multiple genes including ECM components such as collagen, fibrillin, elastin and also TGF-β regulated genes, including TGF-β activated kinase 1/MAP3K7 binding protein 1 (TAB1) [17], [18]. Several of these genes have been already validated in *in vitro* and *in vivo* approaches [17]. In particular, type 1 collagen was shown to be a direct target gene of miR-29a [15], [19]. It was demonstrated that miR-29a downregulated collagen expression in dermal fibroblasts, which resulted in inhibition of ECM deposition. Furthermore, miR-29a was strongly reduced in SSc fibroblasts compared to healthy controls suggesting an important role of this microRNA as a biomarker of fibrogenesis [15].

TGF-β is thought to play a pivotal role in SSc pathogenesis via induction of profibrotic molecules including collagen and TIMP-1 [20]–[22] and by reduction of matrix proteases synthesis [23], [24]. In addition, TGF-β can downregulate miR-29 expression and therefore can indirectly promote ECM accumulation, which is observed in lung and renal fibrosis [18], [25], [26]. Although TGF-β signals via canonical SMAD-dependent pathways and by activation of TGF-β activated kinase 1/MAP3K7 (TAK1) and its binding protein TAB1 [27], there is evidence for a cross-talk and close interference between TAK1 and SMAD pathways upon TGF-β stimulation [28], [29]. Importantly, the binding of TAB1 to TAK1 is essential for TAK1 phosphorylation. TAK1 was shown to be constitutively phosphorylated in fibroblasts isolated from skin lesions of SSc patients [30]. TAK1-deficient murine fibroblasts downregulated TIMP-3 expression, likewise conditional TAK1 KO mice showed reduced dermal thickness and delayed wound closure [30], [31]. These findings imply that TAB1/TAK1 complex plays a crucial role in wound healing and fibrosis development. However, a detailed understanding is still lacking.

In the present study, we demonstrated the ability of miR-29a to prevent fibrosis development in dermal fibroblasts via direct repression of its novel target gene TAB1. This consequently leads to TIMP-1 downregulation and functional collagen breakdown via MMP-1.

## Methods

### Compounds, reagents, and cell culture

Skin biopsy specimens were obtained by punch biopsies from HC and SSc patients. The study was approved by the Health Research Authority (National Research Ethics Service – Sunderland, ethical approval no. 13/NE/0089) and written informed consent was obtained from all patients. Dermal fibroblasts were cultured from skin biopsies as described before [32], and cultured in RPMI (Invitrogen) supplemented with penicillin (100 units/ml), streptomycin (100 µg/ml), L-glutamine (2 mM) (all Sigma) and 10% FSC 37°C in atmosphere containing 5% CO_2_. HeLa cells were cultivated with the same complete RPMI. Additionally, serum-starved fibroblasts were treated with graded doses of (5Z)-7-Oxozeanol (Tocris Science) (TAK-1 inhibitor) in the presence of 5 ng/ml human recombinant TGF-β (Invitrogen) for 24 h to measure TIMP-1 secretion. Cell viability following Oxozeanol treatment was determined using MTS assay (Promega) according to the manufacturer’s recommendations.

### MMP-1, TIMP-1, collagen 1A1, TAB1 mRNA analysis

Total RNA from dermal fibroblasts was extracted using TRIzol reagent (Sigma), according to the manufacturer’s instruction. 500 ng of RNA was reverse transcribed to cDNA using random hexamers and moloney murine leukemia virus reverse transcriptase enzyme (Invitrogen) according to the manufacturer’s protocol. TIMP-1, collagen 1A1 (col1A1), TAB1, MMP-1 and ribosomal 18S expression were analysed using SYBR Green mix. Samples were analysed in triplicate and normalised to the 18S housekeeping gene using the AB7500 (Applied Biosystems) qRT-PCR machine and program. miR-29a expression was normalised to SNORD-25 - endogenous small nuclear RNA control (TaqMan MicroRNA Assays, Applied Biosystems). Relative expression levels to the average healthy control were calculated using the following equation: (2- Delta Delta CT). The sequence of following primers were used; col1A1: forward 5′-CAA GAG GAA GGC CAA GTC GAG G-3′, reverse 5′- CGT TGT CGC AGA CGC AGA T-3′, 18S: forward 5′-GAA TGG CTC ATT AAA TCA GTT ATG G-3′, reverse 5′-TAT TAG CTC TAG AAT TAC CAC AGT TAT CC-3′, MMP-1: forward 5′-AGT GAC TGG GAA ACC AGA TGC TGA-3′, reverse 5′-GCT CTT GGC AAA TCT GGC CTG TAA-3′, TIMP-1: forward 5′-GAC GGC CTT CTG CA ATT CC-3′, reverse 5′-GTA TAA GGT GGT CTG GTT GAC TTC TG-3′. TAB1: forward 5′-ATG AGC TCT TCC GTC TTT CG, reverse 5′-ATC CCC ACC TGC TTG ATC T-3′.

### 3′UTR luciferase reporter assay

The full length of 3′UTR of TAB1 was cloned downstream of firefly luciferase plasmid under the control of SV40 promoter (True Clone, Human Collection, OriGene). HeLa cells were co-transfected with 3′UTR of TAB1 (300 ng) and renilla plasmid (30 ng) using 1.5 µl of Fugene (Promega) transfection reagent. The renilla construct was used to normalise transfection efficiency. Assays for reporter gene activity were performed in duplicate according to Dual-luciferase Pomega’s protocol and analysed using GloMax multi detection system (Promega).

### Transfection experiments

HC or SSc dermal fibroblasts were transfected with synthetic precursor (pre-miR-29, Thermo Scientific) or with negative control (cont-miR - anti-miR-67 Negative Control no 1, Thermo Scientific) at a final concentration of 30 nM using Fugene (Promega). The sequence of cont-miR was designed to be complementary to *C. elegans* transcript, therefore should not interfere with human mRNA. 24 h after transfection, cells were lysed and analysed for expression of type 1 collagen and TIMP-1. In some experiments, HeLa cells or HC dermal fibroblasts were transfected with 500 nM miScript Target protectors (Qiagen). Target protectors were synthesised to be complementary to the seed and flanking regions of 3′UTR of TAB1 thus protecting the target (Table 1).

**Table 1:** Sequence of 3′UTR of TAB1 to which Target protector is complementary (seed regions of 3′UTR of TAB1 are highlighted in bold).

Name	Target binding site sequence	Seed region in 3′UTR TAB1
region A	5′-GCCTTGCATTTTCCTTC**TGGTGCT**GTGAAG-3′	801–807
region B	5′-TTCCAAGAATGCCTCTG**TGGTGCT**GTTTGG-3′	1574–1580

HC dermal fibroblasts were seeded into 24-well plate and transfected using 0.8 µl DharmaFECT transfection reagent (Thermo Scientific) with siRNA against TAB1 (on Target plus Smart pool, Thermo Scientific) or scramble oligo’s at a final concentration of 100 nM. 48 h following transfection, cells were lysed to examine TAB1 protein silencing.

### Functional collagen assay

Collagen gels were prepared as described elsewhere [33]. Briefly, 5×10^4^ HC dermal fibroblasts were seeded on the collagen gels (type 1 collagen from rat tail - C8897, Sigma-Aldrich) and transfected with miR-29a, cont-miR or recombinant TIMP-1 protein was added to the cultures. After 24 h, pictures were taken with the Syngene G-Box imaging system and the gel area was calculated using Image J software.

### Western blot and ELISA analysis

Briefly, 5×10^4^ HC dermal fibroblasts were lysed using Laemmli sample buffer (S3401, Sigma) and subsequently 15 µl of total cell lysates were separated by SDS polyacylamide gel and electrophorised onto nitrocellulose membrane. To detect proteins expression antibodies against human TIMP-1 (ab1827, Abcam), TAB1 (C25E9, Cell Signaling), GAPDH (ab9484, Abcam) and secondary HRP- conjugated (Dako) antibody were used. TIMP-1 and MMP-1 (both R&D Systems) protein concentration in culture supernatants were measured by ELISA according to the manufacturer’s protocol. Signal development was performed using HRP/Streptavidin and OPD substrate (Sigma-Aldrich) at room temperature. Fluorescence was measured using a plate reader (Tecan, Sunrise). Samples were run in duplicate and serial dilution was performed in order to fall within the detection limits of the assay (0–80 ng/ml).

### Statistical and Computational analysis

All data are presented as mean±SEM. The differences between the groups were tested for their statistical significance by non-parametric two tailed T-test using GraphPad Prism (GraphPad Software, San Diego, CA). A *p* value of less than 0.05 was considered statistically significant; *p* values are expressed as follows: ns for not significant; 0.05>*p*>0.01 as*; 0.01>*p*>0.001 as**; *p*<0.001 as***. Computational prediction analyses of mR-29a targeting TAB1 was performed by combining the results of computational prediction algorithms provided by miRDB, miRanda and TargerScanHuman 6.2. These are publically available target prediction databases. Western blot quantification was performed using ImageJ software.

## Results

### Repressed gene expression of type 1 collagen by miR-29a in HC dermal fibroblasts

**Figure 1:**
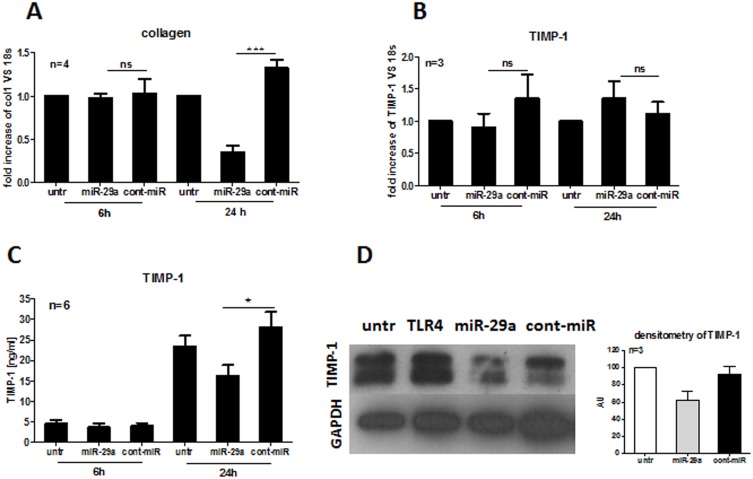
Levels of col1A1 and TIMP-1 mRNA and protein expression after miR-29a transfection. HC dermal fibroblasts were transfected with 30 nM miR-29a, 30 nM non-targeting cont-miR or vehicle only (untr). Col1A1 (A) and TIMP-1 mRNA (B) and TIMP-1 protein secretion (C) were determined after 6 h and 24 h following transfection. TIMP-1 protein level was also determined after 24 h from transfection and following 1 µg/ml TLR4 agonists (LPS) treatment (D). Densitometry analysis of three independent experiments of HC fibroblasts after 24 h from transfection was performed and GAPDH was used as a loading control (D).

HC dermal fibroblasts were transfected with miR-29a or non-targeting control miRNA (cont-miR) 6 and 24 h after transfection, the involvement of miR-29a in ECM remodelling was determined. It is well known that collagen is a target gene for miR-29a expression [15], [16] therefore, to assess the TIMP-1 expression in the presence 30 nM of miR-29a, col1A1 expression was also measured to confirm transfection efficiency of miR-29a. In Figs. 1A and 1B it can be seen that 6 h following transfection, both col1A1 and TIMP-1 mRNA were not repressed by miR-29a. However, miR-29a significantly inhibited col1A1 (*p*<0.001), but not TIMP-1 mRNA after 24 h (Figs. 1A and 1B, respectively). This suggests that col1A1, but not TIMP-1, is a direct target gene of miR-29a. Indeed, following computational analysis we confirmed that the seed region of miR-29a does not have the binding sites to 3′UTR of TIMP-1 mRNA, suggesting that TIMP-1 is an indirect target gene of miR-29a.

### miR-29a repressed the level of TIMP-1 protein in HC dermal fibroblasts

To further define miR-29a activity on the level of TIMP-1 protein, HC dermal fibroblasts were transfected as previously with miR-29a, cont-miR and analysed using ELISA and Western blot. It can be seen that 24 h, but not 6 h (Fig. 1C) following miR-29a transfection, TIMP-1 secretion was significantly downregulated compared to untreated cells (*p*<0.05). Likewise, the level of TIMP-1 protein after 24 h was reduced upon miR-29a, but not in untreated or cont-miR transfected HC dermal fibroblasts assessed by densitometry (Fig. 1D). These findings imply that other protein(s) must be targeted and repressed by miR-29a that also regulates TIMP-1 protein expression. We also found that TLR4 agonist (LPS) induces TIMP-1 production (Fig. 1D), similarly to TLR4-mediated col1A1 induction, which was previously published by Bhattacharyya et al. [34].

### miR-29a alters contractile properties of HC dermal fibroblasts

**Figure 2:**
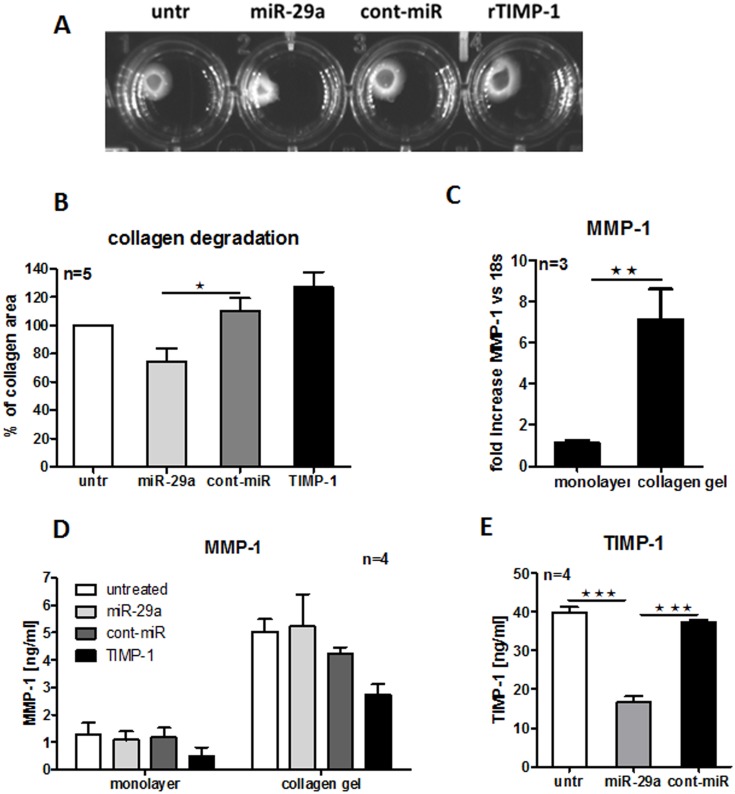
Functional effect of miR-29a on contractile properties of HC fibroblasts. HC dermal fibroblasts were transfected with 30 nM miR-29a, 30 nM cont-miR or stimulated with 100 ng/ml exogenous recombinant TIMP-1 and contractile properties of HC dermal fibroblasts were assessed after 24 h by collagen degradation assay (A) and percentage of collagen gel area was measured (B). MMP-1 mRNA was measured in untreated HC dermal fibroblasts cultivated on monolayers or seeded on collagen gel (C). Secreted MMP-1 level was measured in HC dermal fibroblasts cultivated on monolayers or seeded on collagen gel in the presence of miR-29a, cont-miR or recombinant TIMP-1 (D). Similarly, TIMP-1 secretion was measured in HC dermal fibroblasts seeded on collagen gel in the presence of miR-29a, cont-miR or vehicle only (untr) (E).

To measure the effect of miR-29a on contractile properties of fibroblasts, HC dermal fibroblasts were transfected as previously and tested for contractile properties in a collagen gel degradation assay. This assay is dependent on MMPs, the activity of which results in disintegration of collagen structures [35]–[37]. HC dermal fibroblasts transfected with miR-29a significantly increased gel degradation (Fig. 2A), as the area of collagen gels was reduced by 36% compared to HC dermal fibroblasts transfected with cont-miR (Fig. 2B). We also showed that MMP-1 expression was significantly increased at both the gene and protein level in cells growing on collagen gels compared to HC dermal fibroblasts seeded on standard tissue culture treated plastic (*p*<0.01) (Figs. 2C, 2D). Interestingly, HC dermal fibroblasts cultured on collagen gels secreted higher level of MMP-1 following miR-29a transfection compared to cont-miR, although the difference did not reach statistical significance (Fig. 2D). In order to reduce MMP-1-dependent collagen degradation, we added recombinant TIMP-1 to the media to block catalytic activity of MMPs. As expected, the area of collagen gel was most preserved upon TIMP-1 addition due to MMP-1 inactivation (Figs. 2B and 2D). Furthermore, the level of secreted TIMP-1 significantly decreased in HC dermal fibroblasts transfected with miR-29a compared to cont-miR or untreated cells (*p*<0.001) (Fig. 2E). This suggests that miR-29a not only inhibited secretion of TIMP-1 in HC dermal fibroblasts, but also functionally increased collagen gel degradation by MMP-1 breakdown.

### TAB1 as a novel target of miR-29a

**Figure 3:**
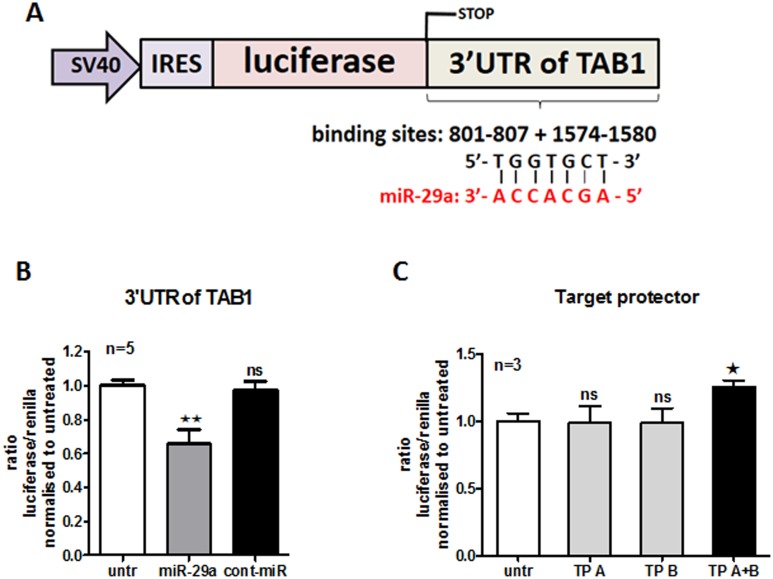
miR-29a binds to 3′UTR of TAB1. HeLa cells were co-transfected with 300 ng 3′UTR TAB plasmid (A) and 30 ng renilla construct in the presence of 30 nM miR-29a, 30 nM cont-miR or vehicle only (untr) (B). Similarly, HeLa cells were co-transfected with 3′UTR TAB construct and renilla in the presence of 500 nM Target Protect binding to seed A - TP A, or seed B - TP B or both - TP A+B (C).

We next investigated which gene(s) can be directly targeted by miR-29a, but can also regulate the TIMP-1 signaling pathway. Following computational prediction analyses using several miRNA target databases including miRDB, miRANDA, HumanScan 6.2, we found that TAB1 has predicted miR-29a target sites. We noticed that miR-29a not only binds to the two regions of 3′UTR of TAB1, but also that TAB1 is activated upon TGF-β [38], a key molecule triggering TIMP-1 production [39]. This suggests that TAB1 might be a good candidate regulating TIMP-1 in the presence of miR-29a. To validate this prediction in *in vitro* experiments, we used a plasmid where 3′UTR of TAB1 was cloned to firefly luciferase construct and transfected into HeLa cells to achieve high transfection efficiency (Fig. 3A), since primary HC fibroblasts were difficult to transfect with exogenous DNA. We used renilla plasmid to normalise 3′UTR-TAB1 plasmid expression in cells transfected with miR-29a and cont-miR or control vehicle (transfection reagent only). As seen in Fig. 3B, the activity of luciferase was significantly decreased, which indicates miR-29a likely targets the 3′UTR of TAB1 resulting in either degradation or inhibition of translation relative to control-miR. Furthermore, we also tested which prediction seed (in position 801–807 refers to A and in position 1574–1580 refer to B, Fig. 2A) plays a crucial role in TAB1 degradation by miR-29a. In order to validate this, we used Target protector’s technique. The RNA sequences of Target protectors are perfectly complementary to miR-29a seed of the 3′UTR of TAB1 and their flanking regions. Thus, Target protectors compete with endogenous miR-29a for seed sequence binding and prevent miRNA/mRNA association due to higher affinity of Target protectors to the TAB1 sequence. This consequently results in hindering gene repression by miR-29a. HeLa cells co-transfected with both Target protectors and 3′UTR of TAB1 plasmid were able to significantly restore luciferase activity (Fig. 3C). However, when HeLa cells were transfected with 3′UTR of TAB1 plasmid and individual Target protector (interfering with either seed A or B), the luciferase activity was similar to control cells. This suggests that individual Target protector was not efficient to prevent repression of luciferase activity by miR-29a via the TAB1 3′UTR degradation, due to the presence of other miR-29a binding sites. Therefore, both Target protectors are required for TAB1 protection from further degradation by endogenous miR-29a in HeLa cells. Of note, the level of endogenous miR-29a was enhanced in HeLa cells compared to HC dermal fibroblasts (data not shown). Therefore, there was a rational use of HeLa cells transfected with Target protector against endogenous miR-29a.

### Target protectors promotes profibrotic phenotype in HC dermal fibroblasts

**Figure 4:**
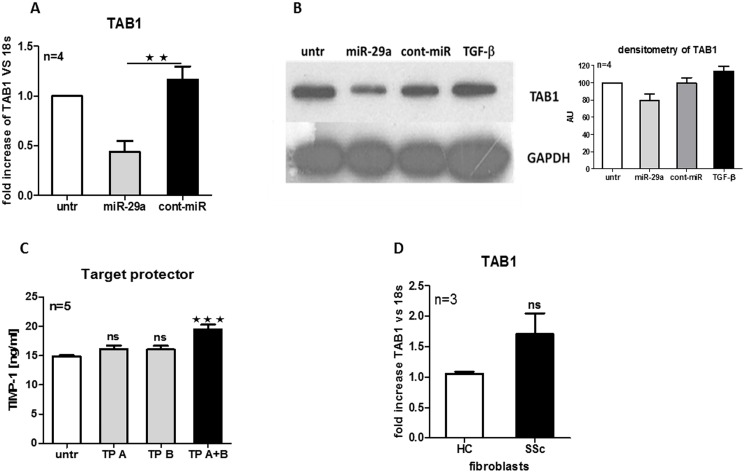
Target protectors induce profibrotic phenotype in HC dermal fibroblasts. HC dermal fibroblasts were transfected with 30 nM miR-29a, 30 nM cont-miR and TAB1 mRNA (A) and protein (B) expression were measured after 24 h. In addition, HC dermal fibroblasts were stimulated with 5 ng/ml TGF-β as a positive control for TIMP-1 induction. Densitometry analysis of four independent experiments was performed and GAPDH was used as a loading control (B). HC dermal fibroblasts were transfected with 500 nM TP A, TP B, or both TP A+B and TIMP-1 secretion was measured after 24 h (C). The basal level of TAB1 mRNA in HC and SSc fibroblasts (D).

In addition, to confirm that miR-29a directly decreases expression of TAB1, HC dermal fibroblasts were transfected with miR-29a and 24 h later TAB1 gene and protein expressions were measured. The expression of TAB1 mRNA was significantly reduced following miR-29a transfection compared to cont-miR (Fig. 4A). Based on densitometry, the level of TAB1 protein was also reduced upon miR-29a transfection, but not in the presence of cont-miR (Fig. 4B). We then asked if suppressing TAB1 degradation by miR-29a affects TIMP-1 secretion. To test this, HC dermal fibroblasts were transfected with both or individual Target protectors and TIMP-1 level was measured by ELISA. As seen previously on luciferase assay, only the presence of both Target protectors allowed for significant derepression of TIMP-1 secretion (*p*<0.001) (Fig. 4C). This suggests that both putative sites of 3′UTR of TAB1 need to hybridise to Target protectors in order to prevent TAB1 degradation by endogenous miR-29a and to promote TIMP-1 production.

In addition, we have measured the basal level of TAB1 in HC and SSc fibroblasts. Although it did not reach statistical significance due to limited number of samples, it can be seen that SSc fibroblasts have 1.7 times higher mRNA level of TAB1 compared to HC fibroblasts (Fig. 4D). We also noticed that profibrotic IL-6 trans-signaling reduced endogenous level of miR-29a in HC fibroblasts (data not shown). This suggests that IL-6 trans-signaling not only promotes profibrotic phenotype via direct collagen and TIMP-1 induction [40], [41], but also can act indirectly by reduction of miR29a levels.

### TAK1/TAB1 inhibitor downregulates TIMP-1 secretion in TGF-β stimulated HC dermal fibroblasts

**Figure 5:**
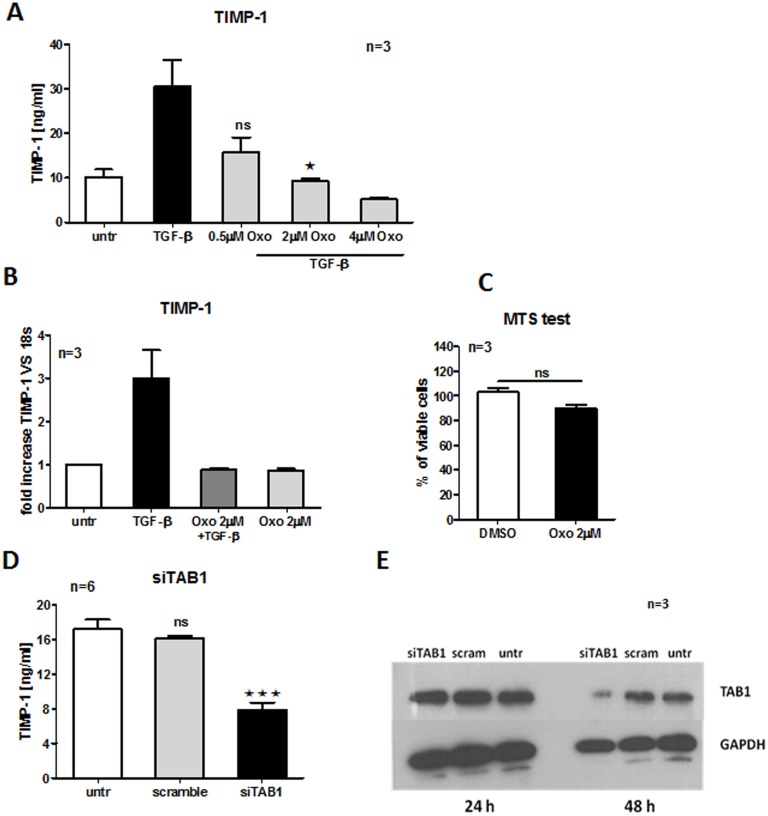
TAK1/TAB1 inhibitor downregulates TIMP-1 secretion in TGF-β stimulated HC dermal fibroblasts. HC dermal fibroblast were pre-treated for 2 h with graded doses of Oxozeanol (TAK1/TAB1 inhibitor) prior to TGF-β stimulation (5 ng/ml) and TIMP-1 secretion and mRNA level were measured (A, B). To assess viability of HC dermal fibroblasts upon 2 µM Oxozeanol treatment, MTS assay was performed (C). HC dermal fibroblasts were transfected with 100 nM siRNA against TAB1 or scrambled oligos and TAB1 protein and TIMP-1 secretion were analysed after 48 h from knockdown (D, E).

We used the chemical agent – (5Z)-7-Oxozeanol (Oxo), which acts as a potent inhibitor of TAB1/TAK1 complexes, to mimic TAB1 downregulation by miR-29a. In particular, Oxo blocks the ATP binding site required for TAK1 kinase activity [42], [43]. Indeed, TIMP-1 secretion was suppressed in the presence of graded doses of Oxo in TGF-β stimulated fibroblasts (Fig. 5A). A concentration of 2 µM of Oxo was the most optimal for TIMP-1 inhibition, but also to keep cells viable, as determined by MTS assay (Fig. 5C). Therefore, we used this concentration in subsequent experiments to measure TIMP-1 mRNA (Fig. 5B). As expected, TIMP-1 mRNA was strongly downregulated in the presence of Oxo in TGF-β stimulated HC dermal fibroblasts. In addition, we measured the level of TIMP-1 in HC dermal fibroblasts transfected with siRNA against TAB1. 48 h following TAB1 knockdown by specific siRNA, confirmed by Western Blotting (Fig. 5E), the level of TIMP-1 protein was reduced (Fig. 5D). Importantly, the level of TIMP-1 in HC dermal fibroblasts transfected with scramble oligos was comparable to control cells. Overall, this implies that blocking TAB1/TAK1 complex activity, either by pharmacological inhibition with Oxo or TAB1 knockdown by siRNA, represses TIMP-1 secretion.

### miR-29a reverses phenotype of SSc dermal fibroblasts

**Figure 6:**
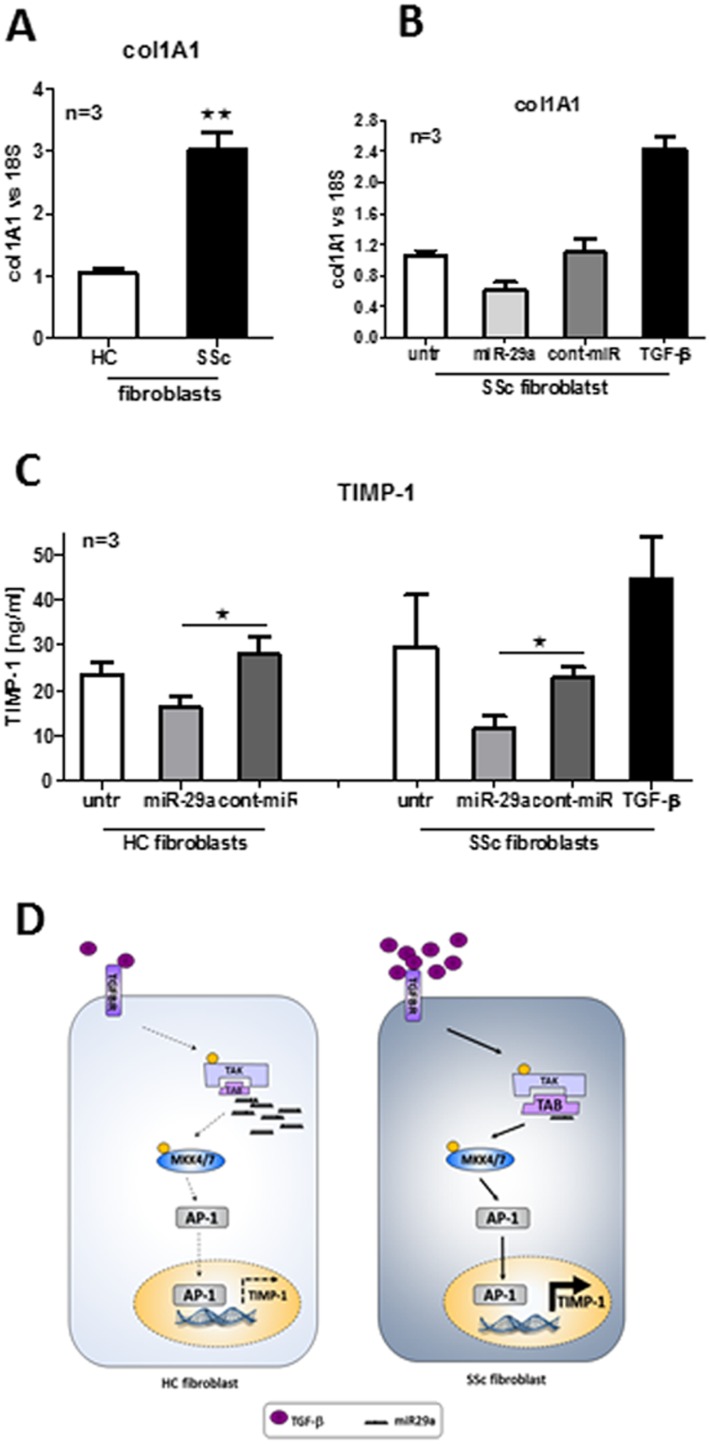
miR-29a reverses phenotype of SSc dermal fibroblasts. The basal level of col1A1 was compared between HC and SSc fibroblasts (A). SSc dermal fibroblasts were transfected with 30 nM miR-29a, 30 nM cont-miR or stimulated with 5 ng/ml TGF-β and col1A1 mRNA level was measured (B). HC and SSc dermal fibroblasts were transfected with 30 nM miR-29a, 30 nM cont-miR or stimulated with 5 ng/ml TGF-β and secreted TIMP-1 was measured (C). Schematic representation of the role of miR-29a in ECM regulation via TAB1 and TIMP-1 suppression in HC and SSc dermal fibroblasts (D). Enhanced activation of TGF-β signaling in SSc dermal fibroblasts compared to HC fibroblasts results in downregulation of miR-29a, which leads to increased expression of miR-29a target gene - TAB1.

Firstly, to confirm the profibrotic phenotype of SSc fibroblasts the levels of col1A1 mRNA and secreted TIMP-1 in untreated HC and SSc fibroblasts were measured. As seen in Fig. 6A the basal level of col1A1 mRNA was significantly elevated (p = 0.0029) in SSc fibroblasts compared to HC. Also secreted TIMP-1 was 1.27 times higher in untreated SSc fibroblasts than HC (Fig. 6C). Finally, we analysed if miR-29a is able to reverse the fibrotic phenotype of SSc dermal fibroblasts. As a positive control for col1A1 and TIMP-1 induction, we stimulated SSc dermal fibroblasts with TGF-β a potent pro-fibrotic cytokine (Figs. 6B and 6C). Indeed, SSc dermal fibroblasts produced more col1A1 and TIMP-1 upon TGF-β treatment. In contrast, miR-29a transfection reduced both col1A1 expression and TIMP-1 secretion. This miR-29a-mediated suppression was specific, as transfection with cont-miR did not have any effect on col1A1 and TIMP-1 synthesis. Overall, based on the above results and previously published findings we hypothesise that the level of miR-29a is reduced in SSc dermal fibroblasts compared to healthy controls due to upregulation of negative regulator of miR-29a including TGF-β and IL-6 trans-signaling [15]. As a consequence of miR-29a reduction, there is excessive production and activation of its target genes such as col1A1 and TAB1. This leads to uncontrolled matrix protein deposition due to increased synthesis of TIMP-1 and ultimately fibrosis progression (Fig. 6D).

## Discussion

SSc is an autoimmune connective tissue disease characterised by fibrosis of skin and internal organs due to accumulation of ECM proteins that consequently leads to organ failure and premature death. Epigenetics is a way of regulating gene expression without a change in the DNA base, which is mostly represented by DNA-methylation, histone modification and miRNA regulation. Emerging evidence suggests a role of miR-29a in SSc and several fibrotic diseases [15], [16], due to its repression of ECM-associated genes like collagen and profibrotic growth factors and their receptors [16], [18], [25].

In the present study, we demonstrated a novel role of miR-29a in fibrogenesis by direct TAB1 targeting resulting in TIMP-1 downregulation. In particular, we showed that TAB1 is a novel *bona fide* target gene of miRNA-29 based on computational algorithms and TAB1 3′UTR luciferase assay. In addition, we showed for the first time that TAB1 regulates TIMP-1 production. It was confirmed by epigenetic (miR-29a and siRNA of TAB1) and chemical (Oxo) approaches, which blocked TAB-1 mediated signaling pathway. Consequently, this resulted in TIMP-1 downregulation, functional collagen degradation and reversal of the profibrotic phenotype of SSc fibroblasts. We also performed a computational analysis which suggests that miR-29a can target 3′UTRs of potent inducers of TIMP-1 including TGF-β2, platelet-derived growth factor subunit B (PDGFB), PDGF subunit C (PDGFC), PDGF receptor subunit B (data not shown). Thus, miR-29a can impact TIMP-1 on multiple different steps.

Firstly, we showed that HC dermal fibroblasts transfected with miR-29a were able to directly reduce col1A1 mRNA and indirectly decrease level of TIMP-1 protein. This TIMP-1 reduction also resulted in an imbalanced ratio between TIMP-1 and MMP-1, in favour of MMP-1. We showed that miRNA-29a-mediated inhibition of TIMP-1 leads to functional collagen breakdown via upregulation of MMP-1 in dermal fibroblasts and therefore prevents matrix protein deposition. The role of miRs in collagen gel disintegration has been also demonstrated by Luna et al [44]. They showed that human trabecular meshwork primary cells transfected with miR-200c increased collagen gel area, due to miR-200c-mediated repression of the main contraction genes including endothelin A receptor and RhoA kinase.

To further elucidate the mechanism of miR-29a-mediated repression of TIMP-1, we used a computational approach to select the best candidate, which could be a target gene of miR-29a and simultaneously regulate TIMP-1 synthesis. Indeed, we found that miR-29a can bind to two seed regions of TAB1 transcript, but also TAB1 is involved in signal transduction upon TGF-β stimulation, a key molecule triggering TIMP-1 production. To address this hypothesis in *in vitro* settings, we used 3′UTR of TAB1 construct that was cloned downstream of firefly luciferase gene and we showed that luciferase activity was decreased by 20 percent in the presence of exogenous miRNA-29a, but not in the presence of control miRNA. However, using specifically designed Target protectors, which block the interaction between endogenous miR-29a and TAB1 transcript due to their higher binding affinity, left TAB1 unaffected. The modified backbone of Target protectors increases their stability and prevents 3′UTR of TAB1 from being loaded into the RNA-induced silencing complex (RISC) and triggering an RNAi response [45]. This was represented by increased luciferase activity in HeLa cells. Importantly, blocking of both seed regions by Target protectors was essential to prevent TAB1 degradation, as the individual seed interference did not rescue 3′UTR of TAB1 to be degraded. Furthermore, HC dermal fibroblasts treated with both Target protectors were able to block endogenous miR-29a interaction with TAB1 resulted in increased secretion of TIMP-1 and thereby in promoting pathogenic phenotype in HC dermal fibroblasts. This gain of function approach strongly suggests that miR-29a binds to both putative sites of 3′UTR of TAB1 and simultaneously plays a role in TIMP-1 regulation.

One of the major kinases involved in the TGF-β signaling cascade is TAK1 and TGF-β is important in fibrosis. It is known that binding of TAB1 is required for TAK1 phosphorylation and further signal transduction [43]. Downstream signaling of TAB1/TAK1 complex plays a central role in regulating MAPK pathways, which subsequently activates several transcription factors. It was shown that NF-κB and activation protein 1 (AP-1) transcription factors respond to TAK1/TAB1 activation and consequently induce proinflammatory and profibrotic genes expression [46]. In particular, it was found that ectopic expression of AP-1 family members resulted in increased expression of TIMP-1 and matrix proteins, suggesting that downstream AP-1 activation is important in TIMP-1 expression and contributes to fibrosis development [47], [48]. However, a clear understanding whether activation of this complex directly leads to TIMP-1 expression is still lacking. In the present study we found that TAB1/TAK1 signaling pathway is involved in regulation of TIMP-1 expression. To confirm that SMAD-independent pathway plays a role in TIMP-1 expression, HC dermal fibroblasts were treated with chemical inhibitor (Oxo) blocking TAK1 activity. As expected, Oxo inhibited TIMP-1 synthesis on both protein and gene levels after TGF-β stimulation. Likewise, HC dermal fibroblasts transfected with siRNA against TAB1 secreted less TIMP-1 following TAB1 siRNA ‘knockdown’. Thus, blocking of TAB1/TAK1 signaling will provide the basis for development more specific targets which will neutralise the effects of TGF-β. Finally, SSc dermal fibroblasts treated with miR-29a displayed an attenuated profibrotic phenotype by downregulation of col1A1 and TIMP-1 when compared to untreated SSc dermal fibroblasts. This data provides an additional aspect that miR-29a is a central player in fibrogenesis in dermal fibroblasts. It is important to remember that SSc fibroblasts have intrinsically reduced levels of miR29a [49]. Molecules that can enhance the expression of miR29a may be useful in reducing the fibrotic response.

## Conclusion

Overall, in the present study, we found that miR-29a directly repressed its novel target gene - TAB1 in dermal fibroblasts, which results in decreased TIMP-1 and increased functional MMP-1 secretion. In contrasts, enhanced expression of miR-29a was able to reverse the profibrotic phenotype of SSc fibroblasts, thus highlighting the potential role of miR-29a in SSc and fibrogenesis in general.

## References

[1] Kikuchi K, Kadono T, Furue M, Tamaki K (1997) Tissue inhibitor of metalloproteinase 1 (TIMP-1) may be an autocrine growth factor in scleroderma fibroblasts. J Invest Dermatol 108:281–284. 9036925 10.1111/1523-1747.ep12286457

[2] Frost J, Ramsay M, Mia R, Moosa L, Musenge E, et al (2012) Differential gene expression of MMP-1, TIMP-1 and HGF in clinically involved and uninvolved skin in South Africans with SSc. Rheumatology (Oxford) 51:1049–1052. 22286923 10.1093/rheumatology/ker367

[3] Young-Min SA, Beeton C, Laughton R, Plumpton T, Bartram S, et al (2001) Serum TIMP-1, TIMP-2, and MMP-1 in patients with systemic sclerosis, primary Raynaud’s phenomenon, and in normal controls. Ann Rheum Dis 60:846–851. 11502611 PMC1753839

[4] Ciechomska M, Cant R, Finnigan J, van Laar JM, O’Reilly S (2013) Role of toll-like receptors in systemic sclerosis. Expert Rev Mol Med 15:e9. 23985302 10.1017/erm.2013.10

[5] Ciechomska M, Huigens CA, Hugle T, Stanly T, Gessner A, et al (2013) Toll-like receptor-mediated, enhanced production of profibrotic TIMP-1 in monocytes from patients with systemic sclerosis: role of serum factors. Ann Rheum Dis 72:1382–1389. 23223421 10.1136/annrheumdis-2012-201958 PMC3711494

[6] Nie QH, Zhang YF, Xie YM, Luo XD, Shao B, et al (2006) Correlation between TIMP-1 expression and liver fibrosis in two rat liver fibrosis models. World J Gastroenterol 12:3044–3049. 16718785 10.3748/wjg.v12.i19.3044 PMC4124379

[7] Yoshiji H, Kuriyama S, Miyamoto Y, Thorgeirsson UP, Gomez DE, et al (2000) Tissue inhibitor of metalloproteinases-1 promotes liver fibrosis development in a transgenic mouse model. Hepatology 32:1248–1254. 11093731 10.1053/jhep.2000.20521

[8] Lovelock JD, Baker AH, Gao F, Dong JF, Bergeron AL, et al (2005) Heterogeneous effects of tissue inhibitors of matrix metalloproteinases on cardiac fibroblasts. Am J Physiol Heart Circ Physiol 288:H461–468. 15650153 10.1152/ajpheart.00402.2004

[9] Macfarlane LA, Murphy PR (2011) MicroRNA: Biogenesis, Function and Role in Cancer. Curr Genomics 11:537–561. 10.2174/138920210793175895 PMC3048316 21532838

[10] Zhu H, Luo H, Zuo X (2013) MicroRNAs: their involvement in fibrosis pathogenesis and use as diagnostic biomarkers in scleroderma. Exp Mol Med 45:e41. 24052166 10.1038/emm.2013.71 PMC3789263

[11] van Rooij E, Purcell AL, Levin AA (2012) Developing microRNA therapeutics. Circ Res 110:496–507. 22302756 10.1161/CIRCRESAHA.111.247916

[12] Patel V, Noureddine L (2012) MicroRNAs and fibrosis. Curr Opin Nephrol Hypertens 21:410–416. 22622653 10.1097/MNH.0b013e328354e559 PMC3399722

[13] Larner-Svensson HM, Williams AE, Tsitsiou E, Perry MM, Jiang X, et al (2010) Pharmacological studies of the mechanism and function of interleukin-1beta-induced miRNA-146a expression in primary human airway smooth muscle. Respir Res 11:68. 20525168 10.1186/1465-9921-11-68 PMC2894768

[14] Pandit KV, Milosevic J, Kaminski N (2011) MicroRNAs in idiopathic pulmonary fibrosis. Transl Res 157:191–199. 21420029 10.1016/j.trsl.2011.01.012

[15] Maurer B, Stanczyk J, Jungel A, Akhmetshina A, Trenkmann M, et al (2010) MicroRNA-29, a key regulator of collagen expression in systemic sclerosis. Arthritis Rheum 62:1733–1743. 20201077 10.1002/art.27443

[16] Peng WJ, Tao JH, Mei B, Chen B, Li BZ, et al (2012) MicroRNA-29: a potential therapeutic target for systemic sclerosis. Expert Opin Ther Targets 16:875–879. 22793265 10.1517/14728222.2012.708339

[17] van Rooij E, Sutherland LB, Thatcher JE, DiMaio JM, Naseem RH, et al (2008) Dysregulation of microRNAs after myocardial infarction reveals a role of miR-29 in cardiac fibrosis. Proc Natl Acad Sci U S A 105:13027–13032. 18723672 10.1073/pnas.0805038105 PMC2529064

[18] Cushing L, Kuang PP, Qian J, Shao F, Wu J, et al (2011) miR-29 is a major regulator of genes associated with pulmonary fibrosis. Am J Respir Cell Mol Biol 45:287–294. 20971881 10.1165/rcmb.2010-0323OC PMC3175558

[19] Du B, Ma LM, Huang MB, Zhou H, Huang HL, et al (2010) High glucose down-regulates miR-29a to increase collagen IV production in HK-2 cells. FEBS Lett 584:811–816. 20067797 10.1016/j.febslet.2009.12.053

[20] Eickelberg O, Kohler E, Reichenberger F, Bertschin S, Woodtli T, et al (1999) Extracellular matrix deposition by primary human lung fibroblasts in response to TGF-beta1 and TGF-beta3. Am J Physiol 276:L814–824. 10330038 10.1152/ajplung.1999.276.5.L814

[21] Leivonen SK, Lazaridis K, Decock J, Chantry A, Edwards DR, et al (2013) TGF-beta-elicited induction of tissue inhibitor of metalloproteinases (TIMP)-3 expression in fibroblasts involves complex interplay between Smad3, p38alpha, and ERK1/2. PLoS One 8:e57474. 23468994 10.1371/journal.pone.0057474 PMC3585359

[22] Kwak HJ, Park MJ, Cho H, Park CM, Moon SI, et al (2006) Transforming growth factor-beta1 induces tissue inhibitor of metalloproteinase-1 expression via activation of extracellular signal-regulated kinase and Sp1 in human fibrosarcoma cells. Mol Cancer Res 4:209–220. 16547158 10.1158/1541-7786.MCR-05-0140

[23] Ihn H (2008) Autocrine TGF-beta signaling in the pathogenesis of systemic sclerosis. J Dermatol Sci 49:103–113. 17628443 10.1016/j.jdermsci.2007.05.014

[24] Derk CT (2007) Transforming growth factor-beta (TGF-beta) and its role in the pathogenesis of systemic sclerosis: a novel target for therapy? Recent Pat Inflamm Allergy Drug Discov 1:142–145. 19075976 10.2174/187221307780979883

[25] Wang B, Komers R, Carew R, Winbanks CE, Xu B, et al (2012) Suppression of microRNA-29 expression by TGF-beta1 promotes collagen expression and renal fibrosis. J Am Soc Nephrol 23:252–265. 22095944 10.1681/ASN.2011010055 PMC3269175

[26] Yang T, Liang Y, Lin Q, Liu J, Luo F, et al (2013) miR-29 mediates TGFbeta1-induced extracellular matrix synthesis through activation of PI3K-AKT pathway in human lung fibroblasts. J Cell Biochem 114:1336–1342. 23238947 10.1002/jcb.24474

[27] Gardner A, Fisher AJ, Richter C, Johnson GE, Moisey EJ, et al (2012) The critical role of TAK1 in accentuated epithelial to mesenchymal transition in obliterative bronchiolitis after lung transplantation. Am J Pathol 180:2293–2308. 22525462 10.1016/j.ajpath.2012.02.022 PMC3366074

[28] Hoffmann A, Preobrazhenska O, Wodarczyk C, Medler Y, Winkel A, et al (2005) Transforming growth factor-beta-activated kinase-1 (TAK1), a MAP3K, interacts with Smad proteins and interferes with osteogenesis in murine mesenchymal progenitors. J Biol Chem 280:27271–27283. 15911626 10.1074/jbc.M503368200

[29] Sano Y, Harada J, Tashiro S, Gotoh-Mandeville R, Maekawa T, et al (1999) ATF-2 is a common nuclear target of Smad and TAK1 pathways in transforming growth factor-beta signaling. J Biol Chem 274:8949–8957. 10085140 10.1074/jbc.274.13.8949

[30] Shi-wen X, Parapuram SK, Pala D, Chen Y, Carter DE, et al (2009) Requirement of transforming growth factor beta-activated kinase 1 for transforming growth factor beta-induced alpha-smooth muscle actin expression and extracellular matrix contraction in fibroblasts. Arthritis Rheum 60:234–241. 19116914 10.1002/art.24223

[31] Guo F, Hutchenreuther J, Carter DE, Leask A (2013) TAK1 is required for dermal wound healing and homeostasis. J Invest Dermatol 133:1646–1654. 23340735 10.1038/jid.2013.28

[32] Normand J, Karasek MA (1995) A method for the isolation and serial propagation of keratinocytes, endothelial cells, and fibroblasts from a single punch biopsy of human skin. In Vitro Cell Dev Biol Anim 31:447–455. 8589888 10.1007/BF02634257

[33] Sheridan CM, Occleston NL, Hiscott P, Kon CH, Khaw PT, et al (2001) Matrix metalloproteinases: a role in the contraction of vitreo-retinal scar tissue. Am J Pathol 159:1555–1566. 11583981 10.1016/S0002-9440(10)62540-0 PMC1850496

[34] Bhattacharyya S, Kelley K, Melichian DS, Tamaki Z, Fang F, et al (2013) Toll-like receptor 4 signaling augments transforming growth factor-beta responses: a novel mechanism for maintaining and amplifying fibrosis in scleroderma. Am J Pathol 182:192–205. 23141927 10.1016/j.ajpath.2012.09.007 PMC3538029

[35] Margulis A, Nocka KH, Wood NL, Wolf SF, Goldman SJ, et al (2009) MMP dependence of fibroblast contraction and collagen production induced by human mast cell activation in a three-dimensional collagen lattice. Am J Physiol Lung Cell Mol Physiol 296:L236–247. 19060229 10.1152/ajplung.90462.2008

[36] Fang Q, Liu X, Al-Mugotir M, Kobayashi T, Abe S, et al (2006) Thrombin and TNF-alpha/IL-1beta synergistically induce fibroblast-mediated collagen gel degradation. Am J Respir Cell Mol Biol 35:714–721. 16858010 10.1165/rcmb.2005-0026OC PMC2643297

[37] Wong WR, Kossodo S, Kochevar IE (2001) Influence of cytokines on matrix metalloproteinases produced by fibroblasts cultured in monolayer and collagen gels. J Formos Med Assoc 100:377–382. 11480246

[38] Kim SI, Kwak JH, Na HJ, Kim JK, Ding Y, et al (2009) Transforming growth factor-beta (TGF-beta1) activates TAK1 via TAB1-mediated autophosphorylation, independent of TGF-beta receptor kinase activity in mesangial cells. J Biol Chem 284:22285–22296. 19556242 10.1074/jbc.M109.007146 PMC2755952

[39] Bujak M, Frangogiannis NG (2007) The role of TGF-beta signaling in myocardial infarction and cardiac remodeling. Cardiovasc Res 74:184–195. 17109837 10.1016/j.cardiores.2006.10.002 PMC1924687

[40] O’Reilly S, Ciechomska M, Cant R, van Laar JM (2014) IL-6 trans signalling drives a STAT3 dependant pathway that leads to hyperactive TGF-beta signalling promoting SMAD3 activation and fibrosis via gremlin. J Biol Chem. 10.1074/jbc.M113.545822 PMC3975039 24550394

[41] Silacci P, Dayer JM, Desgeorges A, Peter R, Manueddu C, et al (1998) Interleukin (IL)-6 and its soluble receptor induce TIMP-1 expression in synoviocytes and chondrocytes, and block IL-1-induced collagenolytic activity. J Biol Chem 273:13625–13629. 9593700 10.1074/jbc.273.22.13625

[42] Safwat N, Ninomiya-Tsuji J, Gore AJ, Miller WL (2005) Transforming growth factor beta-activated kinase 1 is a key mediator of ovine follicle-stimulating hormone beta-subunit expression. Endocrinology 146:4814–4824. 16081641 10.1210/en.2005-0457 PMC1698747

[43] Ninomiya-Tsuji J, Kajino T, Ono K, Ohtomo T, Matsumoto M, et al (2003) A resorcylic acid lactone, 5Z-7-oxozeaenol, prevents inflammation by inhibiting the catalytic activity of TAK1 MAPK kinase kinase. J Biol Chem 278:18485–18490. 12624112 10.1074/jbc.M207453200

[44] Luna C, Li G, Huang J, Qiu J, Wu J, et al (2012) Regulation of trabecular meshwork cell contraction and intraocular pressure by miR-200c. PLoS One 7:e51688. 23272142 10.1371/journal.pone.0051688 PMC3522713

[45] Staton AA, Giraldez AJ (2011) Use of target protector morpholinos to analyze the physiological roles of specific miRNA-mRNA pairs in vivo. Nat Protoc 6:2035–2049. 22134127 10.1038/nprot.2011.423 PMC3779896

[46] Choi ME, Ding Y, Kim SI (2012) TGF-beta signaling via TAK1 pathway: role in kidney fibrosis. Semin Nephrol 32:244–252. 22835455 10.1016/j.semnephrol.2012.04.003 PMC3407377

[47] Hall MC, Young DA, Waters JG, Rowan AD, Chantry A, et al (2003) The comparative role of activator protein 1 and Smad factors in the regulation of Timp-1 and MMP-1 gene expression by transforming growth factor-beta 1. J Biol Chem 278:10304–10313. 12525489 10.1074/jbc.M212334200

[48] Edwards DR, Rocheleau H, Sharma RR, Wills AJ, Cowie A, et al (1992) Involvement of AP1 and PEA3 binding sites in the regulation of murine tissue inhibitor of metalloproteinases-1 (TIMP-1) transcription. Biochim Biophys Acta 1171:41–55. 1420363 10.1016/0167-4781(92)90138-p

[49] Ciechomska M, van Laar JM, O’Reilly S (2014) Emerging role of epigenetics in systemic sclerosis pathogenesis. Genes Immun. 10.1038/gene.2014.44 25030429

